# Nephrologists between power and vulnerability in times of technology

**DOI:** 10.1590/1678-4685-JBN-2018-0011

**Published:** 2018-04-24

**Authors:** José Miguel Viscarra Obregón, Marcio Fabri dos Anjos

**Affiliations:** 1 Universidade Estadual de Maringá, Departamento de Medicina, Disciplinas de Nefrologia e Bioética, Maringá, PR, Brasil.; 2 Centro Universitário São Camilo, Ipiranga, São Paulo, SP, Brasil.; 3 Conselho Regional de Medicina do Estado de São Paulo, Câmara Interdisciplinar de Bioética, São Paulo, SP, Brasil.

**Keywords:** Professional ethics, Vulnerability in health care, Bioethics, Conflicts of interest, Ética profissional, Vulnerabilidade em saúde, Bioética, Conflito de interesses

## Abstract

The doctor-patient relationship is often discussed from the perspective of patient vulnerability. Little attention is given to the vulnerability of nephrologists in their professional practice, a reality often affected by profound cultural transformation arising from technological development. Nephrology is based on research and procedure instrumentalization, both permeated with technology. In addition, the relationship between nephrologists and institutions is governed by market rules. Recent data showed a shortage of new nephrologists and the need to improve the technical training of new professionals, foster the establishment of interventional nephrology, and attract more graduating physicians to this medical specialty. Bioethics offers a different perspective on the issue, since it takes the subjective concerns of medical doctors and the social environments they participate in into consideration in order to enhance their ethical autonomy. These ideas may be discussed as part of undergraduate or specialization programs, thus reinforcing the acknowledgement of vulnerability as a condition and of the relevance of adopting a reflective attitude toward the events of everyday life that interact with the morality of nephrologists, so that risks are adequately faced having bioethical parameters as a reference.

## RESUMO

A vulnerabilidade do paciente é sempre foco de maior atenção quando se analisa a relação médico-paciente. Pouca ênfase é dirigida à vulnerabilidade do nefrologista em relação à própria prática profissional, frequentemente afetada pelas profundas transformações culturais decorrentes do desenvolvimento tecnológico. A atividade da Nefrologia é embasada na pesquisa e na instrumentalização dos procedimentos, ambos permeados pela tecnologia. Além disso, os relacionamentos com instituições são regidos por regras mercadológicas. Dados recentes constatam a escassez de novos nefrologistas, fato que mobiliza a proposta de melhorar a capacitação técnica de novos nefrologistas, favorecendo o estabelecimento da Nefrologia Intervencionista e o implemento durante a graduação médica de ferramentas que aumentem a atratividade por tal especialidade. A Bioética lança um olhar diferente sobre essa questão, pois considera aspectos subjetivos relacionados ao profissional e ao seu entorno social com o intuito de potencializar sua autonomia ética. Essa proposição pode ser considerada durante a graduação e a especialização para o reconhecimento da sua condição de vulnerabilidade e da importância de uma atitude reflexiva que levaria a perceber as incidências cotidianas que agem sobre a moral do nefrologista, para ser possível enfrentar adequadamente os riscos baseado em parâmetros bioéticos.

## INTRODUCTION

Discussions about the professional lives of nephrologists generally focus on patient vulnerability. Attention is rarely devoted to debating the vulnerability nephrologists are faced with. This ethical-reflective study, therefore, aims to explore the latter type of vulnerability as it relates to the cultural transformation introduced by contemporary scientific development. After a few words on transformation, the circumstances interacting with the morality of nephrologists will be identified along with what it takes to tackle the resulting vulnerabilities.

### MEDICINE IN A CONTEXT OF SCIENTIFIC TRANSFORMATION

The changes occurred in the way physicians think are explained by the significant progress medical science has made in the study of the human body and the interplay between myriad factors. On the background are the great transformations engendered since the Renaissance and throughout the eighteenth century, with the formalization of the signs and symptoms of diseases combined with an anatomopathological view of ailments, whose implications are seen to this day^1^ - particularly the idea of what constitutes disease and health, achievements such as immunization, and a fresh perspective on mental health, all of which helped give birth to the clinical rationale in effect today, based on the interpretation of signs exhibited by altered bodily structures to pinpoint the genesis of diseases.

In addition to the ideas physicians had of health and disease and their corresponding bodily expressions, scientific knowledge has also revolutionized the tools used in medical practice. Two types of tools deserve special attention: the ones used to diagnose patients and the ones used to supplement clinical practice. In medical schools, teaching and practice were changed and molded around reductionism, an approach based on the premise that diseases linked to specific organs had to be diagnosed and treated. Based on this idea, the larger areas of medical practice (General Practice, Surgery, Pediatrics, Obstetrics and Gynecology) were subdivided into specialties.^2^

The affirmation of subjective autonomy stemmed from a long process triggered by the revolution of scientific knowledge. The ideals proclaimed in the French Revolution (1789) planted the seed of citizen autonomy and laid the ground for patient autonomy in the context of healthcare - still a strong idea today. Interestingly, when physicians were empowered with new diagnostic tools and clinical resources, the power and influence they once had over their patients vanished, bringing medical paternalism to an end. In addition, their ability to diagnose patients becomes increasingly overshadowed by diagnostic equipment. These events explain why medical work changed profoundly in the twentieth century. Primarily after World War II, being a doctor became a less liberal job and a more technological one - a profession subordinated to the fast pace of scientific progress.

Physicians once enjoyed a significant amount of autonomy, as they saw patients in their offices and had control over their schedules. They could choose to work in hospitals and discuss their fees directly with patients. But decision-making shifted predominantly to the hands of healthcare institutions - hospitals, clinics, outpatient units - and physicians had multiple employment ties in which employer profitability was a primary concern. In addition, the relationship between patients and physicians began to be mediated by other healthcare workers, health management organizations, hospital insurance companies, pharmaceutical companies, and the Government. In other words, medical autonomy was gradually diluted in multiple institutional partnerships and weakened by a brand of technological autonomy. Left with no choice, physicians are inevitably required to accept technology and seek new ways of feeling sufficiently autonomous to make decisions and attain acknowledgement.^3^

The doctor-patient relationship currently unfolds predominantly in hospital settings - in wards, emergency and intensive care units - in which patients with acute manifestations seek care. Once their issues are resolved, the patients are discharged and transferred to outpatient follow-up, usually to be seen by other professionals and thus impeding the creation of a bond between patients and their doctors. This model contrasts with the experience of patients seen in their doctor's office, in which the two are tied for an undetermined length of time and treatment does not result in cure, as diseases are generally of a chronic nature.

### THE IMPACT OF TRANSFORMATION ON NEPHROLOGY

Ever since it was established as a medical specialty in the early 1960s, nephrology has been strongly linked with research and renal replacement therapy (dialysis and kidney transplantation), both examples of the impact science and technology have had in medicine. Dialysis enabled the treatment of patients with acute and chronic kidney disease, a fact that attracted many medical doctors to nephrology for the possibility it offered of applying knowledge generated in basic research to clinical practice and for its uses in diagnosis and treatment. Besides, the related procedures were handled exclusively by nephrologists, namely hemodialysis or peritoneal dialysis; creation of a vascular or peritoneal access for dialysis; percutaneous kidney biopsy for diagnostic purposes and transplant recipient follow-up.

Pioneering nephrologists performed all such procedures, but as complexity and the number of patients grew - chiefly among the elderly and individuals with diabetes - some were reassigned to physicians from other specialties. Radiologists, vascular surgeons and others became essential elements in the work performed by nephrologists. However, nephrology procedures slowly lost their appeal over the years, as technical progress in other areas spurred greater scientific interest and superior income.

Renal replacement therapy is deemed a high-cost medical procedure on account of the massive use of technologically sophisticated equipment and consumables. Primarily the Government assumed the payment for dialysis procedures, which resulted in a significant allocation of funds to dialysis clinics. Nephrologists have had an active role in the economic-administrative aspects of this environment, with varying degrees of involvement: some opted to remain distant from medical procedures to yet receive significant financial compensation, since the payment model for dialysis in effect to this day privileges the payment for materials and medication, including equipment and consumables, over physician fees. Dialysis units grew steadily to meet the care needs of a growing number of individuals with chronic kidney disease. Dialysis clinics - the majority of which privately held - concentrated in medium and large cities located mostly in Southern and Southeastern Brazil.^4^^,^ 5

Newly graduated nephrologists saw the possibility of working in a medical specialty that combined profound clinical knowledge and the technical application of such knowledge. Besides, they also considered the prospect of becoming owners or partners in dialysis clinics as paths to attaining a certain level of financial stability.

In this scenario, the interest in nephrology grew steadily for many years. However, the global economic crisis that shook the world in 2008 and the ensuing administrative adjustments made in healthcare changed things dramatically in nephrology.^6^ The fees and payments for procedures in public and private institutions decreased significantly. These changes and the consequent drop in income have driven nephrologists to seek work in other areas, including intensive care, emergency and outpatient units.

### A NEW GENERATION OF NEPHROLOGISTS

The number of nephrologists entering the labor market has decreased in recent years, as seen in the lack of interest in training programs (internships or residencies) offered at teaching hospitals.^6^ Surveys enrolling graduating medical students on the brink of choosing their specialties have probed into the issue. Patel and Balzer interviewed graduating medical students in the USA and Europe to conclude that students should build a "positive impression" of nephrology throughout their education by having access to more creative methodologies that utilize social media as an educational resource to foster closer integration between theory and practice and show the advantages of longitudinally following patients with chronic diseases.^7^

The considerable decrease in the number of graduating nephrologists deserves attention. Stack et al. looked into the global disproportion between the growing number of patients with chronic kidney disease and the decreasing number of active nephrologists. The authors listed multiple factors contributing to students not opting for nephrology, among which are the lack of demonstration of the positive qualities of nephrology during the course of undergraduate education; procedures once performed solely by nephrologists have been taken over by other medical specialties; absence of a prospect of earning significant financial compensation; uninteresting working environment, repetitive work with little technological innovation and limited alternate procedures; inflexible working hours; little workforce replacement as senior physicians have opted to postpone retirement.^8^

In a paper called "Is Nephrology specialty at risk??", Kalloo et al. borrowed the term "value-added" from the business world to state that a specialty is attractive when it strikes a good balance between technical procedures and medical consultation.9 Given the challenges young physicians choosing nephrology are likely to face, one might assume that the reasons to choose nephrology are connected to the desire to perform technology-based work, achieve financial stability, and improve quality of life, as indicated in the surveys cited above. The 1990s saw the birth of Interventional Nephrology, an attempt to enhance the technical skills of nephrologists - along with their autonomy - and meet the expectations of future specialists. Interventional nephrology has been strongly supported by scientific associations and the academia, and has been geared specifically toward "young nephrologists".^10^^,^ 11

### NEPHROLOGIST EMPOWERMENT: CHALLENGES AND PROSPECTS

The contrast between an early prosperous career for new grads and the less-than-exciting historical development of a medical specialty deemed by many as uninteresting begs questions about the future prospects of a career in nephrology. One ought to reflect and consider matters that extend beyond the job at hand. The challenge of picking a career is not an entirely technical decision, since training - and residency programs in particular - provides for the attainment of proper skill levels. The background question revolves around the attributes that add value to a health professional over and above technical knowledge. According to Fabri, "*Professional practice builds moral personality, since assignments have an enormous power over how one builds oneself and one's relationships with others and the environment. It affects one's identity, health, and self-esteem*".^12^ This idea suggests that attractive and fulfilling careers rise above polarizing financial interests.

Therefore, it is important to analyze the situations experienced by physicians in their daily practice that require perception, critical reflection, and decision-making skills. As described by Schraiber, until the 1960s the practice of medicine as a form of self-employment translated into proximity between doctors and patients, services rendered in the patient's residence or at the doctor's office, and a relationship in which trust was the main element; there, the physician took control of the relational duality of vocation and talent to practice medicine. However, it was not long before scientific progress coupled with capitalism profoundly changed medical practice, now supported by efficient technologies and new traits that significantly impacted the work and profile of medical professionals.

These changes have altered individual and collective expectations around the job, along with the way technical interventions were carried out. Contrast with medicine as a form of self-employment is observed specifically in the technical, organizational, and mercantile arenas. Medicine is now practiced in institutional environments, and the relationship between doctors and patients is mediated through a formal contract; technical skill and accuracy are the sole qualities expected of physicians; patients establish trust with the institution at which they are given medical care; and other stakeholders are involved in providing care to patients.^3^

This emerging environment flourishes in globalized society - known for its fascination with technological development - to directly affect the "means of production of goods, value, meaning, and relationships".^13^ Technological development empowered physicians, but at the same time the fundamental values responsible for their identity and self-esteem were deeply affected, strongly influencing the way they act and feel. It is important to acknowledge the power of the transformations introduced by technological sciences since the last century, not to underrate development, but to wonder about how nephrologists might defend or even reinvent their identity and professional dignity within the new technological scene.

The work of medical doctors is currently governed by the tenets of market economy, which force physicians to deal with countless morally challenging conflictive situations. It is imperative to realize the aggravation of moral conditions. The rules in effect today seek to maximize excellence, a pillar of the business world. The resulting empowerment allows physicians to occupy leading positions in society - a status reserved for the highly competitive, endlessly engaged in the search for up-to-date technical knowledge. This frantic search presupposes the achievement of excellence, a state where error, uncertainty, and doubt may only appear in insignificant levels.

### THE CHALLENGE OF TRAINING NEPHROLOGISTS

The curriculum traditionally offered at medical schools reaffirmed the leading position physicians had in the performance of clinical procedures and found in paternalism its most significant representation. But the prioritization of patient and society demands has led to the development of new educational methods. The majority of medical schools have placed their chips on methods guided by a technological rationale, in which images and virtuality are employed, dismissing completely or relegating to a less important position the learning interpersonal relationships in favor of the acquisition of technical knowledge related to theoretical fundamentals and operational practice. Time is an essential element in teaching and learning activities. A few North-American universities have introduced digital self-learning in medical education, encouraging the development of thinking, memory, and relationship skills among peers and other healthcare workers, in a "team learning" model.^14^^,^ 15 This model is also present in the medical practice of internship programs, in which diagnosis and treatment will no longer be the prerogative of physicians, but rather of the so-called artificial intelligence - robots and cognitive computing - as described in an article on innovation in healthcare published on newspaper *O Estado de São Paulo*:

*Recommending the best course of treatment for each patient and analyzing scans are some of the tasks that will cease to be performed exclusively by physicians - or humans. Progress in areas such as artificial intelligence, cognitive computing, and machine learning has enabled software to read and cross-reference information to levels deemed impossible for the most brilliant experts. However, according to Mariana Perroni, the medical coordinator of Healthcare Transformation at IBM, it is not about replacing healthcare workers, but empowering them. "A specialist would have to study 20 hours a day to keep up with everything that is published in his area. Hard work and good intentions are no longer enough to ensure effective, high-quality care", said Perroni, also an intensive care physician.* 16

Empowerment through technology assumes that medical doctors will no longer undergo classical education by reading manuals or books. Information comes in quickly and easily, as selected by the individual requesting it, reflecting the choice for a shortsighted solution, often void of context. In an environment with nearly no criticism or reflection, alien interests may be represented in the information or knowledge acquired by physicians without going through the analysis and review that would otherwise enable autonomous choice.

Society has also changed significantly in the way it relates to work. The work of physicians is somewhat paradoxical, since "their acts are manual-dependent, yet highly technological; and, at the same time, practice and technological knowledge have been socially appropriated by physicians only".^3^ The social prestige inherent to being a specialist contrasts against the progressive loss of control physicians have experienced in different areas of their profession.

However, one might argue that the strategies for medical education should provide students and teachers with a set of fundamental principles, in which "all action is preceded by reflection",^17^ and prepare future physicians to acknowledge their limitations and consciously accept their vulnerability when faced with the ethical conflicts present in medical work. Attention to patient fragility should not exclude the consideration of physician vulnerability. Fragility is a condition intrinsic to human beings related to our finitude and insufficiency. Direct contact with the world and its contingencies may elicit our vulnerability (vulnus = injury) at any time. Fragility might be thought of as a condition needed in vulnerability.^13^

Medical doctors do not see themselves as fragile or vulnerable; nonetheless, they are exposed daily to risks of all sorts - ethical ones included. It is important that physicians develop critical awareness to understand how their professional context works and the position they occupy before the complex set of factors affecting what they do, namely:

- The legal implications of paid employment and discussions on compensation and fair value; the insufficiency of basic labor rights; working hours not always regulated by the Brazilian Consolidated Labor Laws (CLT);
- The frequent demand from employers to engage in business-to-business instead of employment relationships with physicians, since the first ensues lesser labor risk and lower tax liabilities;
- Gifts offered by pharmaceutical companies in exchange for less than moral or ethical favors;
- The business of a nephrologist requires management skills, assuming that the work of nephrologists is a "business";
- Local conditions to perform the job properly in terms of space, time, and equipment.
- The relationship with health management organizations, in which rules are imposed on physicians (contract-based relationships; acquisition of membership).

Physicians provide care to individuals in situations of fragility for an indefinite period of time; therefore, patient needs might include psychical, social, and cultural demands that require attention and skill before a bond is formed between the parties. Issues such as depression, death, decreased autonomy, and palliative care are often discussed in nephrology. In addition to the multiple dysfunctions presented by their patients, physicians must also deal with the situations arising from the interactions with other healthcare workers involved in treatment.

The ethical and moral assumptions stemmed from the doctor-patient relationship come to fruition only when the parties involved listen to each other. However, this necessary freedom has been the target of external pressures coming from various sources as a result of the changes that Western civilization has undergone in recent centuries, more specifically since the Industrial Revolution. Technology - a symbol of the new era - is gradually taking over the space once dedicated to verbal interaction, a fundamental attribute for the establishment of productive action-oriented dialogue.^18^

## CONCLUSIONS

The lack of interest young physicians have had in nephrology derives from the scarcity of technological procedures performed in this medical specialty and from the disregard with which matters related to the work of nephrologists and the social contexts they interact with are treated when medical students are introduced to the specialties they will eventually choose from.

These issues call for an ex-ante reflection on vulnerability as a human condition in the field of ethics and require that decisions be made based on values acquired previously by medical professionals. Considering these matters is in itself a substantial exercise of citizenship.

Vulnerability does not refer exclusively to the organic fragility inherent to our species. One has to understand that the formative process of personality, morality, and ethics - all indispensable items in one's ability to deal with vulnerability - is not completed in everyone; it relies on a number of variables such as the environment one has lived in since early childhood and the acknowledgement of the participants needed for the formation of a subject's character, considering all interests at play.^17^

Western society is governed by the logic of capitalism. Willingly or not, physicians must be aware of the power that governs, standardizes, and determines the actions influencing medical acts. Taking it as a given, as inescapable fact, strengthens the position of those looking to unilaterally satisfy their interests.^19^ Adopting a political stance in this highly privatized environment is a challenge that enables the implementation of efforts more in tune with the art of medicine, whose essence is based on the bioethical principles of beneficence and nonmaleficence.

It is extremely important to understand how deeply the health-disease complex connects to the social relationships established between patients and healthcare workers under the influence of a hegemonic power. Writing about this topic, Foucault drew attention to "politically active concrete subjects" working on the background of social interactions, particularly in healthcare, submitting physicians to the interests of power holders and promoting exclusion in various degrees and levels. This exclusion affects patients and healthcare workers alike and manifests in various forms and shapes with different degrees of visibility.^20^

Nephrology is a medical specialty known for having close ties with technology and development, in addition to institutional relationships governed by the principles of market economy. Nephrologists are faced with the issues troubling their patients, in situations that often involve risk of death or significant morbidity. Pressure from all sides, conditions imposed by disproportionately stronger stakeholders, and less-than-exciting prospects of enjoying a fairer and more dignifying professional life are elements present in the daily life of nephrologists.

By acknowledging vulnerability, nephrologists strengthen their autonomy and take a firmer stance in their interactions with other stakeholders. As subjects aware of their condition, newly graduated physicians may take over the status of apprentices of nephrology with a more thorough understanding of the experiences they are likely to go through in their careers.
